# Machine Learning-Driven GLCM Analysis of Structural MRI for Alzheimer’s Disease Diagnosis

**DOI:** 10.3390/bioengineering11111153

**Published:** 2024-11-15

**Authors:** Maria João Oliveira, Pedro Ribeiro, Pedro Miguel Rodrigues

**Affiliations:** CBQF—Centro de Biotecnologia e Química Fina—Laboratório Associado, Escola Superior de Biotecnologia, Universidade Católica Portuguesa, Rua de Diogo Botelho 1327, 4169-005 Porto, Portugal; s-mjboliveira@ucp.pt (M.J.O.); s-pmsbribeiro@ucp.pt (P.R.)

**Keywords:** Alzheimer’s disease, mild cognitive impairment, structural magnetic resonance imaging, gray-level co-occurrence matrix, classical machine learning

## Abstract

Background: Alzheimer’s disease (AD) is a progressive and irreversible neurodegenerative condition that increasingly impairs cognitive functions and daily activities. Given the incurable nature of AD and its profound impact on the elderly, early diagnosis (at the mild cognitive impairment (MCI) stage) and intervention are crucial, focusing on delaying disease progression and improving patients’ quality of life. Methods: This work aimed to develop an automatic sMRI-based method to detect AD in three different stages, namely healthy controls (CN), mild cognitive impairment (MCI), and AD itself. For such a purpose, brain sMRI images from the ADNI database were pre-processed, and a set of 22 texture statistical features from the sMRI gray-level co-occurrence matrix (GLCM) were extracted from various slices within different anatomical planes. Different combinations of features and planes were used to feed classical machine learning (cML) algorithms to analyze their discrimination power between the groups. Results: The cML algorithms achieved the following classification *accuracy*: 85.2% for *AD* vs. *CN*, 98.5% for *AD* vs. *MCI*, 95.1% for *CN* vs. *MCI*, and 87.1% for *all* vs. *all*. Conclusions: For the pair *AD* vs. *MCI*, the proposed model outperformed state-of-the-art imaging source studies by 0.1% and non-imaging source studies by 4.6%. These results are particularly significant in the field of AD classification, opening the door to more efficient early diagnosis in real-world settings since MCI is considered a precursor to AD.

## 1. Introduction

Alzheimer’s disease (AD) is a progressive neurodegenerative disease characterized by memory loss and multiple cognitive impairments (memory, attention, concentration, speech, and thinking, among others) [1]. AD represents one of the most expensive, lethal, and burdening diseases of this century. This deterioration leads to alterations in an individual’s behavior, personality, and functional capacity, impeding their ability to perform activities of daily living [2].

The World Health Organization (WHO) estimates that more than 55 million people (8.1% of women and 5.4% of men over 65 years) are living with dementia, a number that could reach 78 million by 2030 and nearly triple to 139 million by 2050 [3]. Within this context, AD assumes a prominent position, accounting for approximately 60% to 80% of all instances of dementia [4]. Given that the predominant risk factor for dementia is advancing age, the ongoing rise in life expectancy and demographic aging further amplifies the probability of individuals developing this condition. Thus, AD directly impacts cognitive abilities, and neurocognitive function presents significant challenges, particularly in populations with increasing life expectancies [5]. These are clear indicators that this dementia-related disorder will pose considerable challenges to public health and elderly care systems across the world [6].

AD follows a progressive disease sequence that extends from an asymptomatic phase with biomarker evidence of AD through minor cognitive and/or neurobehavioral changes to, ultimately, AD dementia [4]. This translates into the AD continuum, where three broad phases exist: preclinical Alzheimer’s disease, mild cognitive impairment (MCI) due to AD, and dementia due to AD, also called Alzheimer’s dementia [7].

During the preclinical phase, individuals exhibit signs of AD pathology without apparent cognitive or functional deterioration, comprising a long asymptomatic phase where day-to-day routines remain unperturbed [4]. In turn, MCI identifies individuals who do not have dementia but do have biomarker evidence of Alzheimer’s brain changes, as well as new but subtle deficits in cognition. Difficulties in memory, language, and thought processes arise when the brain reaches a point where it can no longer offset the detrimental impact and loss of neurons [7,8]. Within the subset of individuals experiencing MCI, around 15% undergo progression to dementia within two years. Additionally, approximately one-third of those with MCI develop dementia associated with AD within five years [7]. The AD dementia stage itself can be divided into mild, moderate, and severe, depending on the magnitude of the disease’s manifestation and the resulting decline in the patient’s level of independence.

Despite clinical measures being available for the diagnosis of AD progression, the process is notably time-intensive. The first steps in diagnosis take action when a patient presents symptoms consistent with the early stages of AD. In clinical practice, a two-stage process is often employed. This involves an initial “triage”, typically conducted through primary care, where it is essential to review the clinical background and family history and exclude possible reversible causes of cognitive impairment, such as depression or vitamin, hormone, and electrolyte deficiencies, through routine examinations, namely blood analyses [4]. One of the most frequently reported manifestations involves memory-related symptoms. Therefore, clinical evaluations are performed to gauge the existence of cognitive and functional impairments. A cognitive assessment that can be efficiently conducted (within 10 min), albeit with certain constraints, is the Mini-Mental State Examination [4]. This assessment scores memory and language impairments on a scale from 0 to 30, with lower scores meaning more severe impairments. As for the second stage, once an initial assessment is undergone and the presence of cognitive impairment is confirmed, specialists employ clinical supplementary evaluations to ascertain the underlying causes of the impairment and validate the diagnosis with the identification of neurodegeneration and biomarker presence through magnetic resonance imaging (MRI), positron emission tomography (PET), electroencephalogram (EEG), or cerebrospinal fluid (CSF) analysis. Table 1 shows the state-of-the-art AD detection using a comprehensive comparison of studies of the ADNI and other imaging and non-imaging databases.

Although initially mainly used to rule out other causes of cognitive impairment, MRI has demonstrated positive predictive value for AD now that new imaging analysis modalities have emerged [31]. Structural MRI directs its focus towards the physical modifications occurring in the brain regions affected by the neuropathology of AD, such as atrophy and changes in tissue characteristics [31,32]. For instance, hippocampal degeneration is correlated with increased relevant biomarker deposition, leading to a smaller hippocampal volume (HV) in AD patients showing declined cognitive function. Thus, HV serves as a recommended biomarker, indicating neuronal damage, which supports the diagnosis of AD in the presence of clinical symptoms. Notably, the accelerated atrophy of the hippocampus is a reliable predictor for an increased likelihood of conversion from MCI to AD [32]. Despite the absence of molecular specificity, sMRI is regarded as secure and effective, providing valuable measures of brain atrophy. These metrics encapsulate the accrued neuronal damage, which, in turn, is a direct determinant of the clinical state [32].

While clinical measures and diagnostic tools are available for the assessment of AD progression, the process is notably time-intensive. Moreover, accurately evaluating the disease’s development can be challenging until the symptoms become overt, even for experts. Therefore, early detection has become a focal point for the scientific community. Early diagnosis not only prompts precautionary measures but also has the potential to mitigate the disease’s non-curable adverse effects on the patient’s daily life in the foreseeable future [5]. With this in mind, our work focuses on developing an automated diagnostic tool for discrimination between MCI and AD using sMRI texture features. Our primary objectives include the following:

- To introduce the utilization of 22 sMRI gray-level co-occurrence matrix features for the characterization of AD activity;
- To strengthen the differentiation between healthy control, MCI, and AD patients by systematically comparing the synergistic power of GLCM features extracted across different sMRI planes;
- To thoroughly evaluate the discriminatory performance of the GLCM features by employing an extensive set of machine learning models.

## 2. Materials and Methods

**Figure 1:**
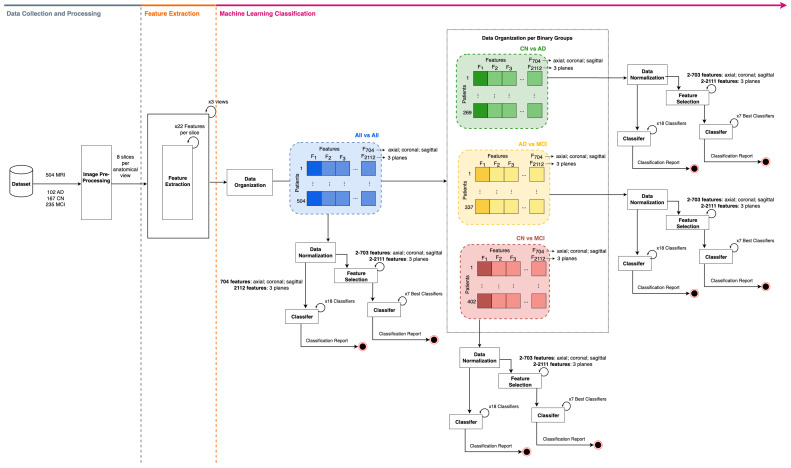
Methodology workflow diagram.

The methodology conducted in this study is illustrated in Figure 1. It is divided into three main phases: data collection/pre-processing, feature extraction, and machine learning classification. This research was conducted using a MacBook Pro 14 (Apple Inc., Cupertino, CA, USA) equipped with an M3 Pro chip featuring an 11-core CPU, a 14-core GPU, 18 GB of RAM, and 512 GB SSD.

### 2.1. Dataset Description

The Alzheimer’s Disease Neuroimaging Initiative (ADNI) database provided the brain MRI datasets used in this work. Specifically, the first phase of ADNI1 is available for download at http://adni.loni.usc.edu (accessed on 14 September 2024) [33]. The ADNI1 MRI procedure used dual-echo T2-weighted and T1-weighted sequences to provide consistent longitudinal structural imaging on 3T and 1.5T scanners. Each patient’s average scan time was about forty-five minutes per session. Many imaging vendors, such as Siemens, GE Healthcare, and Philips Healthcare, adopt this uniform procedure. Strict quality control methods were applied to every examination to find and eliminate images judged unsuitable due to subject mobility or inadequate anatomical coverage. A total of 504 images from individual participants in the ADN1 acquisitions, representing a range of diagnostic groupings, were included in the dataset: 167 represent healthy controls, 235 patients with MCI, and 102 patients diagnosed with AD. It is important to note that not all MCI patients were β-amyloid positive in this dataset.

Table 2 presents details regarding each group’s demographic characteristics.

### 2.2. Image Pre-Processing

#### 2.2.1. Data Cleaning

From the different ADN1 collections, the images were selected so as to obtain a single image for a specific subject, selecting the one associated with the most recent date.

One of the objectives of ADNI1 included evaluating structural MRI metrics at both 1.5T and 3T to assess whether the strength of the magnetic field significantly influenced the quantitative measures that are critical for AD. The researchers concluded that the performance with both field strengths was similar, with neither presenting significant disadvantages over the other [34]. Thus, 3T and 1.5T scans were both used to maximize the number of single subjects’ images, as the differences in the image quality between 1.5T and 3T MRI are minimal. This approach enabled the expansion of the study groups’ sizes for the present investigation.

#### 2.2.2. Skull Stripping

**Figure 2:**
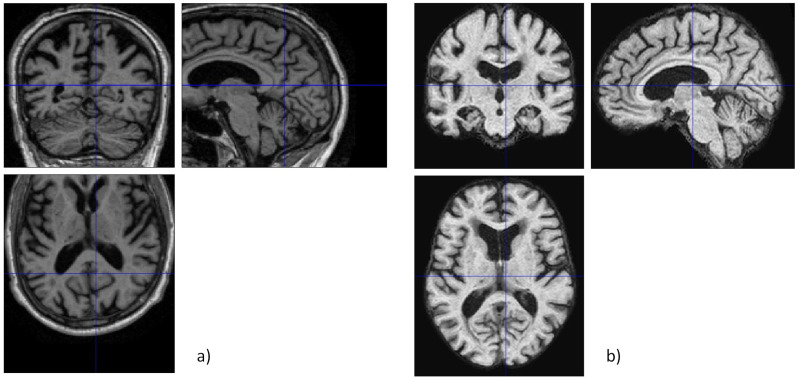
Skull stripping process in SPM: (**a**) original image; (**b**) processed image.

Although some image corrections were already provided by the ADNI, such as the correction of image geometry distortion or intensity non-uniformity, the selected images were loaded into the MATLAB 2023b software, where a set of structural pre-processing steps were performed with the help of the SPM12 (Statistical Parametric Mapping) package (freely available online at https://www.fil.ion.ucl.ac.uk/spm/ (accessed on 15 September 2024), which is designed for the analysis of brain imaging data sequences. These structural steps were applied according to [35,36] and included segmentation, skull stripping, and normalization. Segmentation is essential for the differentiation of tissue types and the calculation of the deformation matrix, which dictates the location of the result on a specific brain coordinate template. Extracting the skull region is a crucial step in brain segmentation assignments for clinical analysis, and it is known as skull stripping. Effective skull stripping becomes essential because of the brain’s complex anatomical structure and intensity variations in MRI. This process’s precision and efficiency are paramount for accurate diagnostics [37]. The normalization step at this point was important to normalize the image to a standard space, defining the boundaries around the brain from a set origin. An example of the resulting image from the structural steps is provided in Figure 2.

#### 2.2.3. Slicing and Final Pre-Processing Steps

The resulting images underwent decomposition into 2D slices representing three distinct anatomical planes: coronal, sagittal, and axial. Employing a MATLAB 2023b routine, eight slices were generated from each image for every anatomical plane. This specific number of slices was chosen due to computational constraints, as it balanced detailed anatomical representation with a manageable data volume and processing time. Additionally, it is essential to note that these slices were consistently extracted from identical positions across all images to ensure uniformity in the data analysis.

Subsequently, all acquired 2D slices were converted from the initial Neuroimaging Informatics Technology Initiative (NIfTI) format to Portable Network Graphics (PNG) and subjected to transformation. The transformation involved resizing the input images to dimensions of 156 × 156. This resizing significantly reduced the computational time during the feature extraction phase. Moreover, the use of square images streamlined the subsequent analysis. Following image resizing, a normalization process was implemented for each slice. Normalization scales the pixel values in an image from a predefined range (0 to 255) to a standardized range between 0 and 1 [38].

### 2.3. Feature Extraction

For each subject, eight images per anatomical view, resulting in a total of 24 images per subject, and a cumulative total of 12,096 images for the entire dataset consisting of 504 study participants were employed for feature extraction. From each image, 22 texture features were computed. The gray-level co-occurrence matrix (GLCM) method was used for this.

#### Texture Analysis via GLCM

Using MATLAB 2023b and the function *graycomatrix* (available in the MATLAB Image Processing Toolbox), it was possible to generate a matrix by determining the frequency of occurrences of a pixel. In this work’s context, the function output consisted of multiple GLCMs, specified to the *graycomatrix* function by an array of offsets, which defined pixel relationships of varying directions and distances. Specifically, the array utilized defined four offsets [01;−11;−10;−1−1], representing the distance between a pixel of interest and its neighboring pixels in four distinct directions: horizontal, vertical, and two diagonals. Employing multiple directions facilitates the more sensitive characterization of the texture within the image, encompassing texture patterns across various orientations [39].

Additionally, the number of gray levels used was 8, the default value for numeric images, which is the case for MRI images. This determines the discrete intensity levels considered in GLCM computation and, ultimately, the size of the GLCM (8 × 8). Moreover, this selection balances the capture of textural intricacies and the management of the computational demands [39].

Although the function *graycoprops* (available in the MATLAB Image Processing Toolbox) successfully extracts four statistics specified in the properties of the GLCMs—contrast, correlation, energy, and homogeneity—which were used, a few more parameters were also computed according to [40]. Each feature output vector had four dimensions corresponding to the four-direction offset array. Table 3 provides a comprehensive overview of all extracted features [40]. To fully understand the data presented, the specific notations used to describe the matrix entries and associated calculations are provided in the Notation part of the manuscript.

All of the extracted feature information was organized into matrices, with subjects as rows and features as columns, categorized by group (AD, CN, MCI) and anatomical view (axial, coronal, sagittal, and combined views). A comprehensive matrix was also constructed by combining the information from all three groups for all anatomical views.

The z-score normalization method was applied to each matrix to ensure the consistent treatment of each feature. In MATLAB, z-score normalization [41] is performed by column, which standardizes each feature across the subjects.

### 2.4. Classification Framework

The normalized matrices were subsequently uploaded to Python (version 3.9.12) to execute a set of machine learning models and generate discrimination reports.

#### 2.4.1. ML Discrimination Between Study Groups—No Feature Selection Approach

The model’s performance in discriminating between study groups (*AD* vs. *CN*, *AD* vs. *MCI*, *CN* vs. *MCI*, and *all* vs. *all*) was evaluated using eighteen selected *Scikit-learn* ML models, employing a hold-out method. Each algorithm processed either 2112 features when utilizing data from all three anatomical planes (22 features extracted, each with (×) 4 offsets from (×) 8 slices (×) per 3 anatomical planes, for each group comparison) or 704 features when analyzing each plane individually (22 features extracted, each with (×) 4 offsets from (×) 8 slices (×) per 1 anatomical plane, for each group comparison).

In the hold-out method, the dataset was divided randomly into a *train* and *test* set. Specifically, 80% of the data was allocated for training, and the remaining 20% was used for testing. The training set was utilized for model training, while the test set was used to evaluate the model’s performance on unseen data.

Table 4 provides details of each classifier used and the corresponding hyperparameters. For this purpose, some configurations were created, but the default hyperparameters provided by *Scikit-learn* were also used [42].

#### 2.4.2. ML Discrimination Between Study Groups—Feature Selection Approach

Following an analysis of the outcomes from the previous phase (no feature selection approach), a subset of seven classifiers demonstrating superior Accuracy was selected for classification between the study groups using a feature selection approach. In the evaluation of the model’s capability to discriminate between the study groups (using the same hold-out process), feature selection, utilizing the F-score algorithm [43], was systematically conducted by incrementally adding to the model’s entries one feature per iteration, ranging from 2 to 2111 features (combined plane variant) or from 2 to 703 features (per plane). The feature selection process was strictly performed using the training data to prevent any data leakage assumptions between training and testing.

#### 2.4.3. Classification Metrics

The performance evaluation of the proposed model was conducted using ten metrics: accuracy, specificity, precision, recall, F1-score, AUC (area under the curve), and Gmean.

Accuracy represents the number of correctly classified classes concerning all cases and can be defined as

(1) $$Accuracy=\frac{TP+TN}{TP+TN+FP+FN}\times 100\%$$

where TP, TN, FP, and FN are, respectively, the true positives, true negatives, false positives, and false negatives [42].

Precision, also known as the positive predictive value, indicates the proportion of correctly classified positive cases among all cases predicted as positive. It can be defined as [42]

(2) $$Precision=\frac{TP}{TP+FP}\times 100\%$$

Recall, also known as sensitivity, represents the proportion of correctly predicted positive cases among the total number of actual positive cases, being defined as [42,44]

(3) $$Recall=\frac{TP}{TP+FN}\times 100\%$$

Specificity is the negative class version of the recall and denotes the rate of negative samples that are correctly classified. The specificity ranges from 0 to 1, where 1 indicates the perfect prediction of the negative class, and 0 means that all negative class samples are incorrectly predicted. It is defined as [44]

(4) $$Specificity=\frac{TN}{FN+TN}\times 100\%$$

The F1-score is the harmonic average between the recall and precision, penalizing extreme values of either [44]. The corresponding equation is defined as [42]

(5) $$F1-Score=\frac{2\times Precision\times Recall}{Precision+Recall}\times 100\%$$

The geometric mean (Gmean) is a metric that evaluates the balance in performance across all classes. A higher Gmean value indicates a reduced risk of overfitting, which means that the model has been trained in a specific way to the training sample, compromising its ability to generalize to new data [45]. The Gmean is mathematically defined as [42]

(6) $$Gmean=\sqrt{Recall\times Specificity}\times 100\%$$

The area under the curve (AUC) of the receiver operating characteristic (ROC) curve assesses a model’s ability to discriminate between positive and negative classes. It achieves this by comparing the true and false positive rates across various classification thresholds. The AUC values range from 0 to 1, with a perfect classifier yielding 1 and a random classifier yielding 0.5. The AUC concisely measures a model’s performance, enabling straightforward comparison between models and evaluations in scenarios with class imbalances [42].

## 3. Classification Results

The obtained discrimination report metrics for both variations (without and with feature selection) are documented in the following subsections.

### 3.1. Discrimination Results Without Feature Selection

The discrimination results when utilizing the complete feature set are presented in Table 5, Table 6, Table 7 and Table 8. The best results are highlighted in blue.

- *CN* vs. *AD*: Referring to Table 5, the highest accuracy of 85.2% was achieved using information from the three planes, obtained through the cML algorithms *LinSVC* or *OvsR*. The lowest classification accuracy was 66.7%, obtained using the axial plane.
- *AD* vs. *MCI*: From Table 6, the highest classification accuracy of 98.5% was achieved from the three planes using the *LogReg* classifier. The axial plane exhibited the lowest classification Accuracy, recording a value of 64.7%.
- *CN* vs. *MCI*: As seen in Table 7, the most notable classification outcome was 88.9%, achieved from the coronal plane using the ExTreeC cML algorithm. The axial plane yielded the least favorable outcome, demonstrating accuracy of 56.8%.
- *all* vs. *all*: Table 8 shows that the most significant classification accuracy of 82.2% was achieved through the cML algorithms *LinSVC* or *OvsR*. The minimum accuracy attained was 49.5%, again from the axial plane.

### 3.2. Discrimination Results with Feature Selection

The discrimination results obtained using the F-score method for feature selection are presented in Table 9, Table 10, Table 11 and Table 12. The best results are highlighted in blue.

- *CN* vs. *AD*: Referring to Table 9, the highest classification accuracy of 85.2% was achieved utilizing the sagittal plane, incorporating 491 selected features and employing the *ExTreeC* classifier. The lowest classification accuracy was 72.2% using the axial plane.
- *AD* vs. *MCI*: From Table 10, the highest classification accuracy of 98.5% was attained through the ML algorithms *LinSVC* or *OvsR*, using 3 planes and 1379 features. The lowest classification accuracy attained was 80.9% using the axial plane.
- *CN* vs. *MCI*: From Table 11, the peak accuracy value of 95.1% was obtained using 1129 features selected from the three planes and the ExTreeC classifier. The minimum accuracy reached was 67.9% from the axial plane.
- *all* vs. *all*: As indicated in Table 12, the classification presented the highest accuracy, 87.1%, for the three planes using 1366 features selected and the *LinSVC* classifier. The axial plane demonstrated the lowest classification accuracy, registering 55.4%.

## 4. Discussion

We divide this section into two subsections to provide a comprehensive discussion. The first subsection compares the approach with no feature selection to the feature selection approach. The second subsection compares the results of the present work with the state-of-the-art results.

### 4.1. No Feature Selection Approach vs. Feature Selection Approach

Checking the results presented in Table 5, Table 6, Table 7, Table 8, Table 9, Table 10, Table 11 and Table 12, it is possible to see that feature selection overall enhances the classification results.

Regarding the accuracy, the FS procedure outperformed the non-FS procedure by 0.9% for the pair CN vs. AD, 5.4% for CN vs. MCI, and 3.8% for all vs. all.

The recall also showed significant improvements from non-FS to FS, indicating a reduction in false negatives (FN). Improving the recall scores is essential since failing to detect positive cases of AD or misclassifying its stages can have severe consequences.

The F1−score also increased when applying the FS procedure, reflecting a balanced enhancement in both identifying more real positive cases (higher recall) and making fewer false positive errors (higher precision). In the AD diagnosis context, where classification errors must be minimized, this improvement demonstrates the robustness of the model when applying FS.

The AUC score also improved, enhancing the model’s ability to accurately classify patients into AD, MCI, or CN. This improvement aids in better evaluating the model’s performance, particularly in scenarios with class imbalances, such as the one involving MCI in the present study.

The above coincides with the known advantages of feature selection—identifying and retaining only the essential or relevant features for the classification task, culminating not only in better classification results but also in reducing the dimensionality of the data, improving the overall performance [46].

Focusing on the feature selection results, the two result values above 90% correspond to the pairs *CN* vs. *MCI* and *AD* vs. *MCI*. This is of high importance because, firstly, there is no active cure for AD at present. As MCI is considered a transitional stage between normal cognitive aging and AD, monitoring individuals diagnosed with MCI allows for the early detection of those who are more likely to progress to AD, enabling interventions and treatments to be initiated earlier. Thus, distinguishing between CN and MCI, as well as AD and MCI, can be helpful for clinicians and caregivers in the early development of personalized treatment plans tailored to the individual’s stage of cognitive decline.

The *CN* vs. *MCI* and *AD* vs. *MCI* groups show consistently good performance across all metrics reported. For the pair *CN* vs. *MCI*, the high specificity (96.2%) demonstrates the model’s exceptional capability in correctly identifying CN individuals (the negative class), which highlights its efficiency in avoiding false positive diagnoses, ensuring that individuals without MCI are rarely misclassified, and avoiding inappropriate medical interventions for those not affected by MCI. Furthermore, the Gmean of 94.6% ensures that the specificity is well balanced with the sensitivity (recall), indicating that the model effectively manages classification errors (FP and FN). Additionally, the AUC score of 94.6% signifies the model’s robust ability to discriminate between the CN and MCI classes, performing well even in scenarios where the class proportions are not even. Concerning the pair *AD* vs. *MCI*, the precision of 100.0%, paired with the recall of 94.1%, suggests a highly reliable model that is excellent in correctly identifying MCI cases without falsely labeling AD patients as MCI. The Gmean of 99.0% is highly relevant in this case, enhancing the confidence that, even in the presence of class imbalances, which happens for this study pair, the model does not disproportionately favor one class over the other.

Typically, the pair *CN* vs. *AD* would exhibit the highest classification performance since these groups present the most significant anatomical differences in the brain [12]. This is also supported by the significant differences between the MMSE scores reported in Table 2, which are related to the subject’s level of symptomatology. Nevertheless, it is important to note that accurately distinguishing between cognitively normal individuals and those with AD presents significant challenges due to the complex and heterogeneous nature of the condition [47]. Despite these challenges, this work has demonstrated considerable strength in precision, showing that, when the model predicts AD, these predictions are correct about 88.2% of the time, which is crucial to avoid the consequences of FP, such as unnecessary testing or treatment for individuals incorrectly diagnosed with AD. Moreover, the Gmean of 86.0% suggests that the model maintains a good balance between recall and specificity, essential in minimizing both types of classification errors, indicating that the model performs satisfactorily well in identifying both CN and AD conditions with a low error rate.

Regarding the *all* vs. *all* pair, although the multi-class classification proved to be challenging, the model’s capability is affirmed by key metrics. Alongside the accuracy of 87.1%, the model achieved an F1-score of 87.3%, indicating a well-tuned model that not only captures most positive cases (recall) but also ensures that its predictions of the positive class are accurate (precision), minimizing the risk of harmful FP. Concurrently, the specificity (92.7%) and Gmean (90.0%) show that the model correctly identifies negative cases and provides balanced performance across various class distinctions, suggesting a reduced risk of overfitting.

Examining the outcomes across various study planes in Section 3.2, it is evident that the most successful results were obtained when utilizing the brain’s three-dimensional representation (using all three planes). This outcome was expected since combining the coronal, axial, and sagittal planes provides a holistic perspective on the brain, enabling the more precise visualization of critical structures for AD detection, namely the cerebral cortex, ventricles, and hippocampus, which are among the principal regions implicated in AD pathology [48]. Incorporating data from all three anatomical planes can offer complementary features, beneficial for classification endeavors, as evidenced by research findings indicating that multi-view models tend to exhibit superior accuracy compared to single-view models [49].

### 4.2. Study Results vs. State-of-the-Art Results

**Figure 3:**
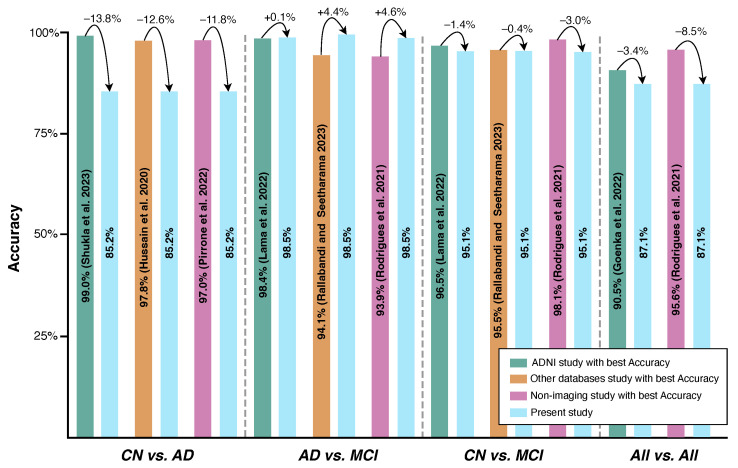
State-of-the-art comparison with the present study (best accuracy). For reference: (Shukla et al. 2023 [11]), (Hussain et al. 2020 [22]), (Pirrone et al. 2022 [26]), (Lama et al. 2022 [14]), (Rallabandi and Seetharama 2023 [23]), (Rodrigues et al. 2021 [27]), and (Goenka et al. 2022 [17]).

While acknowledging that, in medicine, the accuracy may not fully capture the balance between recall and specificity, the following comparison between the present study’s results and state-of-the-art results will primarily focus on the accuracy to check the model’s performance, as it enables a more direct comparison; see Table 1 and Figure 3.

Focusing on the findings from the ADNI database, the following should be noted.

- Not all state-of-the-art studies attempted the three binary classifications conducted in the present work.
- Only four state-of-the-art studies performed multi-classification—*all* vs. *all*. The present study outperformed the work of [12,20] by 11.8% and 16.1%, respectively. However, the present study showed 3.4% lower accuracy compared to the findings in [17].
- The present work did not outperform the state-of-the-art work concerning the pair *CN* vs. *AD*, where it was surpassed by margins of accuracy ranging from 2.3% [16] to 13.8% [11].
- In terms of the pair *CN* vs. *MCI*, the proposed model outperformed the majority of the consulted state-of-the-art sMRI-based studies [9,12,15,16,17,18,19,20] by 9.5%, 6.9%, 2.2%, 15.9%, 17.1%, 22.7%, 18.2%, and 5.9%, respectively. The one that yielded better performance than the current work did so by a maximum of 1.4% accuracy [14].
- The pair that showed the higher classification accuracy, *AD* vs. *MCI*, successfully outperformed not only all sMRI-based studies but also the ones related to fMRI and PET, enhancing the importance of the present work’s findings. Higher results have been obtained with differences ranging from 0.10% [14] to 25.8% [19] regarding accuracy. Although the classes were imbalanced in the present work, due to the high number of MCI patients when compared with AD patients, the accuracy attained was accompanied by a Gmean value of 99.0%, suggesting that the model generalizes well, avoiding overfitting. This is a significant finding, opening the door to more efficient early diagnosis in real-world settings since MCI is considered a precursor to AD.

When comparing the present work with other sMRI-based studies that used other databases rather than the ADNI, some conclusions can be obtained.

- The performance of the present work in distinguishing between *CN* and *AD* did not surpass that of the state-of-the-art studies. Specifically, it was outperformed with accuracy margins ranging from 2.8% [21] to 12.6% [22].
- Compared with the study in [23], the present study shows a gain of 4.4% regarding the accuracy for the *AD* vs. *MCI* pair, but a loss of 0.4% for the *CN* vs. *MCI* pair.

Finally, comparing the present results with those obtained from other examination modalities, the proposed model was able to achieve the following:

- It surpassed the achieved accuracies for the *AD* vs. *MCI* pair when compared with the studies in [26,27] by 15.5% and 4.6%, respectively;
- It surpassed the reported accuracies for *CN* vs. *MCI* by 18.1% [28] and by 0.1% [26], although it showed a loss of 3.0% when compared with [27];
- It outperformed the study in [26] by 12.1%, but was outperformed by the algorithms employed in the studies in [27,28] by 8.5% and 2.9% in terms of accuracy, respectively; nevertheless, it was unable to outperform the state-of-the-art accuracies for the pair *CN* vs. *AD*, being surpassed by 11.8% [26], 10.8% [27], and 9.8% [28];
- It underperformed with regard to the Rodrigues et al., 2021 study [27] for the multi-classification set (all vs. all), with differences of about 8.5%.

Although enriching, these comparisons must be carefully analyzed, because different signals, image databases and sources, feature selection methods, and classification means were employed in the state-of-the-art studies. It should also be noted that most state-of-the-art studies did not report other classification metrics, focusing only on the accuracy, which can be misleading. The proposed model presents a comprehensive set of metrics, demonstrating its accuracy in correctly classifying instances and effectively discriminating between classes, making it ready for implementation. Furthermore, this work introduces a model closer to market implementation using a hold-out validation method, which splits the dataset into training and testing subsets. This approach leverages the dataset’s considerable size to simulate the performance on unseen data, providing a realistic assessment of the model’s generalization capabilities, unlike most state-of-the-art methods presented in Table 1, which rely on cross-validation.

## 5. Conclusions

Alzheimer’s disease poses significant challenges due to its incurability and challenging diagnostic process. Early detection is of great interest, offering the possibility to delay the onset of debilitating symptoms that erode patients’ quality of life and shorten their lifespans. This work aimed to introduce an artificial intelligence model that can detect AD at the stages of CN, MCI, and AD itself using sMRI. By analyzing pre-processed brain images, 22 features computed from the gray-level co-occurrence matrix were extracted. Initially, machine learning algorithms were trained using the complete feature set, followed by a refinement step where only the most discriminant and compatible features were utilized, thereby incorporating a feature selection process. The findings demonstrated that feature selection consistently improved the model performance, resulting in heightened accuracies and enhancements in other relevant classification metrics to support more reliable and adequate decision-making in practical settings. Consequently, the algorithms achieved the following classification accuracies: 85.2% for *CN* vs. *AD*, 98.5% for *AD* vs. *MCI*, 95.1% for *CN* vs. *MCI*, and 87.1% for *all* vs. *all*. For the pair *AD* vs. *MCI*, the proposed model outperformed all of the consulted state-of-the-art imaging studies by 0.1% and the non-imaging source studies by 4.6%, as shown in Figure 3. It should be noted that Figure 3 is designed to illustrate both the improvements and limitations in terms of the discrimination accuracy of our method compared with the current state-of-the-art approaches, while acknowledging that a direct comparison and conclusions should always be considered carefully given the varied methodologies employed, database sizes, source data, and the potential influence of the applied processes’ margins of error.

Considering that most studies did not present results for *all* vs. *all* classification, the proposed model added this variant and performed well. Additionally, the use of a wide array of classification metrics, rather than only focusing on the accuracy, offers a broader and more profound view of the model’s performance, boosting our confidence in it. Therefore, the potential of this work in facilitating the diagnosis of AD in real-world circumstances, particularly in the early stages, is reinforced.

Although the results suggest that the proposed model can aid in the diagnostic process, providing medical practitioners with an additional tool for more confident decision-making, future work should focus on balancing the number of subjects within the classified groups and expanding the overall dataset. This will ensure more reliable generalization and, thus, a more robust model that is better prepared for real-world application. Furthermore, it could be beneficial to understand the impact of applying other forward-based feature selection methods instead of the F-score, e.g., Pearson’s correlation, linear discriminant analysis, ANOVA, or chi-squared tests, to maximize the discriminative performance of the cML models. Finally, it could also be interesting to extend and test the capability of the presented algorithm solution to detect preclinical AD pathologies in CN and cases of subjective cognitive impairment (SCI), which is another key medical need in the field of early AD diagnosis.

## Figures and Tables

**Table 1:** Comprehensive comparison of studies on ADNI and on other databases.

Study	Database	# of Subjects	Exam Modality	Features Extracted	Best Classifier	Feature Selection	Validation Approach	Accuracy
Within ADNI Database								
[9]	ADNI	1200	sMRI	Spatial Information	Lightweight Neural Network	Not Applied	Hold-out	CN vs. AD—95.0% CN vs. MCI—85.6% AD vs. MCI—87.5%
[10]	ADNI	1296	sMRI	Hierarchical Patterns, Textures, and Other Structural Features	2D CNN (ResNet-50v2)	Not Applied	Cross-validation	CN vs. AD—91.2% All vs. All—90.3%
[11]	ADNI	600	sMRI	Statistical Features	Ensemble LR_SVM	K-Best	Cross-validation	CN vs. AD—99.0% CN vs. MCI—96.0% AD vs. MCI—85.0%
[12]	ADNI	89	sMRI	Statistical and Textural Features	BagT FGSVM QSVM SubKNN	F-score	Cross-validation	CN vs. AD—93.3% CN vs. MCI—88.2% AD vs. MCI—87.7% All vs. All—75.3%
[13]	ADNI	96	fMRI	Graph Theoretical Measures	Linear SVM	MRMR	Cross-validation	CN vs. AD—96.8%
[14]	ADNI	95	fMRI	Correlation Matrix Between Different ROIs	SL-RELM	Adaptive Structure Learning	Cross-validation	CN vs. AD—95.4% CN vs. MCI—96.5% AD vs. MCI—98.4%
[15]	ADNI	2116	sMRI	Multi-Scale and Hierarchical Transformed Features	CNN (PKG-Net)	Not Applied	Cross-validation	CN vs. AD—94.3% CN vs. MCI—92.9% AD vs. MCI—92.1%
[16]	ADNI	178	sMRI	Cortical Volumetric Features	Fully Connected Neural Network	PCA	Cross-validation	CN vs. AD—87.5% CN vs. MCI—79.2% AD vs. MCI—83.3%
[17]	ADNI	381	Amyloid PET	3D Slice-Based Features	3D CNN	Not Applied	Hold-out	CN vs. AD—92.9% CN vs. MCI—78.0% AD vs. MCI—87.2% All vs. All—90.5%
[18]	ADNI	798	sMRI	Spatial Features	Ensemble based on 2D CNN	Not Applied	Hold-out	CN vs. AD—90.4% CN vs. MCI—72.4% AD vs. MCI—77.2%
[19]	ADNI	179	sMRI	Cortical Surface Labels	DNN	Not Applied	Cross-validation	CN vs. AD—85.2% CN vs. MCI—76.9% AD vs. MCI—72.7%
[20]	ADNI	801	sMRI	Voxel-Level GM Volume Density	CNN Ensemble Model	Not Applied	Cross-validation	CN vs. AD—92.8% CN vs. MCI—89.2% AD vs. MCI—81.5% All vs. All—71%
Present Study	ADNI	504	sMRI	Statistical and Textural Features	ExTreeC LinSVC OvsR	F-Score	Hold-out	CN vs. AD—85.2% CN vs. MCI—95.1% AD vs. MCI—98.5% All vs. All—87.1%
Different Imaging Databases								
[21]	AIBL	1455	sMRI	Voxel-Based Features	SVM	Not Applied	Cross-validation	CN vs. AD—88.0%
[22]	OASIS	416	sMRI	Model Components	12-layer CNN	Not Applied	Hold-out	CN vs. AD—97.8%
[23]	OASIS	1098	MRI-PET Fusion	MRI-PET Feature Maps	Inception–ResNet CNN Model	CNN-Based Ranking	Hold-out	CN vs. AD—95.9% CN vs. MCI—95.5% AD vs. MCI—94.1%
[24]	OASIS	77	MRI	CNN-Based Features	KNN	ROC-Based Ranking	Cross-validation	CN vs. AD—95.0%
[25]	FHS	102	sMRI	Disease Probability Maps	CNN	Not Applied	Hold-out	CN vs. AD—76.6%
[25]	NACC	582	sMRI	Disease Probability Maps	CNN	Not Applied	Hold-out	CN vs. AD—81.8%
Non-Imaging Modalities								
[26]	IRCCS	105	EEG	Frequency Domain Features	KNN	Information Gain Filter	Cross-validation	CN vs. AD—97.0% CN vs. MCI—95.0% AD vs. MCI—83.0% All vs. All—75.0%
[27]	University Hospital Center of São João	38	EEG	Cepstral and Lacstral Distances	ANN	KW test	Cross-validation	CN vs. ADM—96.0% CN vs. MCI—98.1% ADM-ADA vs. MCI—93.9% All vs. All—95.6%
[28]	University Hospital Center of São João	38	EEG	Relative Power, Spectral Ratios, Maxima, Minima, and Zero Crossing Distances	ANN	KW test	Cross-validation	CN vs. AD—95.0% CN vs. MCI—77.0% AD vs. MCI—83.0% All vs. All—90.0%
[29]	ADNI	623	Biomarkers	SNPs, Gene and Clinical Data	SVM	Mutual Information	Cross-validation	CN vs. MCI/AD—95.0%
[30]	DementiaBank	269	Speech Signals	Statistical and Non-Linear Parameters	LogReg	Not Applied	Cross-validation	CN vs. AD—85.2%

**Table 2:** Database demographics overview.

Group	# of Subjects	Age Average ± SD	Gender F | M		MMSE Average ± SD
CN	167	77.9 ± 5.33	82	85	29.1 ± 1.16
MCI	235	77.0 ± 7.04	77	158	25.0 ± 4.22
AD	102	77.0 ± 7.33	50	52	18.9 ± 6.12

**Table 3:** Feature description.

Feature	Formula	Description
Autocorrelation	$\sum_{i}\sum_{j}\left(ij\right)p\left(i,j\right)$	Measures the magnitude of the fineness and coarseness of the texture
Contrast	$\sum_{n=0}^{N_{g}-1}n^{2}\left\{\sum_{i=1}^{N_{g}}\sum_{j=1}^{N_{g}}p\left(i,j\right)|\left|i-j\right|=n\right\}$	Measures the local variations between pixels
Correlation 1	$\frac{HXY-HXY1}{max\left\{HX,HY\right\}}$	Estimates the combined probability occurrence of the indicated pixel pairs
Correlation 2	$\frac{\sum_{i}\sum_{j}\left(ij\right)-\mu_{x}\mu_{y}}{\sigma_{x}\sigma_{y}}$	Estimates the combined probability occurrence of the indicated pixel pairs
Cluster Prominence	$\sum_{i}\sum_{j}{\left(i+j-\mu_{x}-\mu_{y}\right)}^{4}p\left(i,j\right)$	Measures the skewness and asymmetry of the GLCM
Cluster Shade	$\sum_{i}\sum_{j}{\left(i+j-\mu_{x}-\mu_{y}\right)}^{3}p\left(i,j\right)$	Measures the skewness and uniformity of the GLCM
Dissimilarity	$\sum_{i}\sum_{j}\left|i-j\right|\cdot p\left(i,j\right)$	Measures the local intensity variation, defined as the mean absolute difference between neighboring pairs
Energy	$\sum_{i}\sum_{j}p{\left(i,j\right)}^{2}$	Specifies the sum of squared elements in the GLCM
Entropy	$-\sum_{i}\sum_{j}p\left(i,j\right)log\left(p\left(i,j\right)\right)$	Assesses the randomness of an intensity image
Homogeneity 1	$\sum_{i}\sum_{j}\frac{p(i,j)}{1+\left|i-j\right|}$	Measures the nearness of the distribution of elements in the GLCM to the GLCM diagonal
Homogeneity 2	$\sum_{i}\sum_{j}\frac{1}{1+{\left(i-j\right)}^{2}}p\left(i,j\right)$	Measures the nearness of the distribution of elements in the GLCM to the GLCM diagonal
Maximum Probability	$\max_{i,j}\left(p(i,j)\right)$	Occurrence of the most predominant pair of neighboring intensity values
Variance	$\sum_{i}\sum_{j}{\left(i-\mu \right)}^{2}p\left(i,j\right)$	Measures the distribution of neighboring intensity level pairs about the mean intensity level in the GLCM
Sum Average	$\sum_{i=2}^{2N_{g}}ip_{x+y}\left(i\right)$	Measures the relationship between the occurrences of pairs with lower intensity values and the occurrences of pairs with higher intensity values
Sum Variance	$\sum_{i=2}^{2N_{g}}{\left(i-f_{16}\right)}^{2}p_{x+y}\left(i\right)$	Measures groupings of voxels with similar gray-level values
Sum Entropy	$-\sum_{i=2}^{2N_{g}}p_{x+y}\left(i\right)log\left\{p_{x+y}\left(i\right)\right\}$	Measures neighborhood intensity value differences
Difference Variance	$\mathrm{variance}\;\mathrm{of}\;p_{x-y}$	Measures the heterogeneity that places higher weights on differing intensity level pairs that deviate more from the mean
Difference Entropy	$\sum_{i=0}^{N_{g}-1}p_{x-y}\left(i\right)log\left\{p_{x-y}\left(i\right)\right\}$	Measures the randomness/variability in the neighborhood intensity value differences
Information Measure of Correlation 1	$\frac{HXY-HXY1}{max\left\{HX,HY\right\}}$	Assesses the correlation between the probability distributions of *i* and *j*
Information Measure of Correlation 2	${\left(1-exp\left[-2.0\left(HXY2-HXY\right)\right]\right)}^{\frac{1}{2}}$	Assesses the correlation between the probability distributions of *i* and *j*
Inverse Difference Normalized	$\sum_{k=0}^{N_{g}-1}\frac{p(x-y(k))}{1+\left(\frac{k}{N_{g}}\right)}$	Measures the local homogeneity of an image
Inverse Difference Moment Normalized	$\sum_{k=0}^{N_{g}-1}\frac{p(x-y(k))}{1+\left(\frac{k^{2}}{N_{g}^{2}}\right)}$	Measures the local homogeneity of an image

**Table 4:** *Scikit-learn* ML classifier configurations.

Classifier	Hyperparameters
*GaussianProcessClassifier (GauPro)*	1.0×RBF(1.0)
*LinearSVC (LinSVC)*	Default parameters
*SGDClassifier (SGD)*	max_iter: 100, tol: 0.001
*KNearestNeighborsClassifier (KNN)*	Default parameters
*LogisticRegression (LogReg)*	solver: “lbfgs”
*LogisticRegressionCV (LogRegCV)*	cv: 3
*BaggingClassifier (BaggC)*	Default parameters
*ExtraTreesClassifier (ExTreeC)*	n_estimators: 300
*RandomForestClassifier (RF)*	max_depth: 5, n_estimators: 300, max_features: 1
*GaussianNB (GauNB)*	Default parameters
*DecisionTreeClassifier (DeTreeC)*	max_depth: 5
*MLPClassifier (MLP)*	α: 1, max_iter: 1000
*AdaBoostClassifier (AdaBoost)*	Default parameters
*QuadraticDiscriminantAnalysis (QuaDis)*	Default parameters
*OnevsRestClassifier (OvsR)*	random_state: 0
*LightGBMClassifier (LGBM)*	Default parameters
*GradientBoostingClassifier (GradBoost)*	Default parameters
*SGDClassifier (SGD)*	max_iter: 100, tol: 0.001

**Table 5:** Classification metrics for the pair *CN* vs. *AD*.

*CN* vs. *AD*	Classifier	Accuracy	Recall	Precision	Specificity	F1-Score	AUC	Gmean
**3 Planes**	*LinSVC/OvsR*	85.2%	77.3%	85.0%	85.3%	81.0%	83.9%	85.1%
**Axial Plane**	*DeTreeC*	66.7%	10.5%	66.7%	66.7%	18.2%	53.8%	66.7%
**Coronal Plane**	*LinSVC*	70.4%	52.4%	64.7%	73.0%	57.9%	67.1%	68.7%
**Sagittal Plane**	*LogRegCV*	79.6%	57.9%	78.6%	80.0%	66.7%	74.7%	79.3%

**Table 6:** Classification metrics for the pair *AD* vs. *MCI*.

*AD* vs. *MCI*	Classifier	Accuracy	Recall	Precision	Specificity	F1	AUC	Gmean
**3 Planes**	*LogReg*	98.5%	94.1%	100.0%	98.1%	97.0%	97.1%	99.0%
**Axial Plane**	*BaggC*	64.7%	40.0%	40.0%	75.0%	40.0%	57.5%	54.8%
**Coronal Plane**	*ExTC*	94.1%	73.3%	100.0%	93.0%	84.6%	86.7%	96.4%
**Sagittal Plane**	*LinSVC/OvsR*	95.6%	95.2%	90.9%	97.8%	93.0%	95.5%	94.3%

**Table 7:** Classification metrics for the pair *CN* vs. *MCI*.

*CN* vs. *MCI*	Classifier	Accuracy	Recall	Precision	Specificity	F1	AUC	Gmean
**3 Planes**	*LinSVC/OvsR*	80.2%	74.4%	82.9%	78.3%	78.4%	80.0%	80.5%
**Axial Plane**	*BaggC*	56.8%	40.0%	50.0%	60.4%	44.4%	54.8%	54.9%
**Coronal Plane**	*ExTreeC*	88.9%	83.8%	91.2%	87.2%	87.3%	88.5%	89.2%
**Sagittal Plane**	*ExTreeC*	82.7%	80.6%	75.8%	87.5%	78.1%	82.3%	81.4%

**Table 8:** Classification metrics for the pair *all* vs. *all*.

*All* vs. *All*	Classifier	Accuracy	Recall	Precision	Specificity	F1	AUC	Gmean
**3 Planes**	*LinSVC/OvsR*	82.2%	82.2%	83.0%	89.9%	81.9%	89.8%	86.2%
**Axial Plane**	*BaggC*	49.5%	49.5%	48.4%	74.5%	48.1%	62.2%	60.0%
**Coronal Plane**	*ExTreeC*	65.3%	65.3%	62.5%	84.2%	63.6%	86.6%	71.8%
**Sagittal Plane**	*ExTreeC*	68.3%	68.3%	68.1%	85.6%	66.3%	86.4%	76.2%

**Table 9:** Classification metrics for the pair *CN* vs. *AD*-FS.

*CN* vs. *AD*	# of Features	Classifier	Accuracy	Recall	Precision	Specificity	F1	AUC	Gmean
**3 Planes**	943	*LogRegCV*	77.8%	70.8%	77.3%	78.1%	73.9%	77.1%	77.7%
**Axial Plane**	204	*OvsR*	72.2%	42.9%	75.0%	71.4%	54.5%	66.9%	73.2%
**Coronal Plane**	533	*LinSVC/OvsR*	79.6%	65.2%	83.3%	77.8%	73.2%	77.8%	80.5%
**Sagittal Plane**	491	*ExTreeC*	85.2%	71.4%	88.2%	83.8%	78.9%	82.7%	86.0%

**Table 10:** Classification metrics for the pair *AD* vs. *MCI*-FS.

*AD* vs. *MCI*	# of Features	Classifier	Accuracy	Recall	Precision	Specificity	F1	AUC	Gmean
**3 Planes**	1379	*LinSVC/OvsR*	98.5%	94.1%	100.0%	98.1%	97.0%	97.1%	99.0%
**Axial Plane**	8	*BaggC*	80.9%	50.0%	69.2%	83.6%	58.1%	71.0%	76.1%
**Coronal Plane**	645	*ExTreeC*	91.2%	80.0%	95.2%	89.4%	87.0%	88.8%	92.3%
**Sagittal Plane**	199	*ExTreeC*	95.6%	95.8%	92.0%	97.7%	93.9%	95.6%	94.8%

**Table 11:** Classification metrics for the pair *CN* vs. *MCI*-FS.

*CN* vs. *MCI*	# of Features	Classifier	Accuracy	Recall	Precision	Specificity	F1	AUC	Gmean
**3 Planes**	1129	*ExTreeC*	95.1%	93.1%	93.1%	96.2%	93.1%	94.6%	94.6%
**Axial Plane**	5	*ExTreeC*	67.9%	58.8%	62.5%	71.4%	60.6%	66.6%	66.8%
**Coronal Plane**	606	*ExTreeC*	86.4%	75.0%	93.1%	82.7%	83.1%	85.3%	87.7%
**Sagittal Plane**	509	*ExTreeC*	88.9%	75.9%	91.7%	87.7%	83.0%	86.0%	89.7%

**Table 12:** Classification metrics for the pair *all* vs. *all*-FS.

*All* vs. *All*	# of Features	Classifier	Accuracy	Recall	Precision	Specificity	F1	AUC	Gmean
**3 Planes**	1366	*LinSVC*	87.1%	87.1%	87.7%	92.7%	87.3%	94.7%	90.0%
**Axial Plane**	694	*BaggC*	55.4%	55.4%	57.0%	75.2%	55.2%	73.8%	64.7%
**Coronal Plane**	495	*ExTreeC*	75.2%	75.2%	73.6%	89.5%	73.4%	87.2%	81.1%
**Sagittal Plane**	608	*ExTreeC*	71.3%	71.3%	68.6%	89.5%	67.7%	89.3%	78.1%

## Data Availability

The data used in the preparation of this article were obtained from the Alzheimer’s Disease Neuroimaging Initiative (ADNI) database (http://adni.loni.usc.edu, accessed on 21 September 2024). As such, the investigators within the ADNI contributed to the design and implementation of the work and/or provided data but did not participate in the analysis or writing of this report. A complete list of the ADNI investigators can be found at http://adni.loni.usc.edu/wp-content/uploads/how_to_apply/ADNI_Acknowledgement_List.pdf, (accessed on 21 September 2024).

## Notation

p(i,j)	(i,j)th entry in a normalized gray-tone
	spatial dependence matrix.
$N_{g}$	Number of distinct gray levels in the
	quantized image
$\mu_{x}$, $\mu_{y}$	Mean gray level intensities of $p_{x}$ and $p_{y}$,
	respectively
$\sigma_{x}$, $\sigma_{y}$	Standard deviations of $p_{x}$ and $p_{y}$, respectively
H(X)	Entropy of $p_{x}$
H(Y)	Entropy of $p_{y}$
$p_{x}\left(i\right)=\sum_{j=1}^{N_{g}}p\left(i,j\right)$	Marginal row probabilities
$p_{y}\left(j\right)=\sum_{i=1}^{N_{g}}p\left(i,j\right)$	Marginal column probabilities
$p_{x+y}\left(k\right)=\sum_{i=1}^{N_{g}}\sum_{j=1}^{N_{g}}p\left(i,j\right)$	$\mathrm{where}\;i+j=k\;\mathrm{and}\;k=2,3,\ldots ,2N_{g}.$
$p_{x-y}\left(k\right)=\sum_{i=1}^{N_{g}}\sum_{j=1}^{N_{g}}p\left(i,j\right)$	$\mathrm{where}\;|i-j|=k\;\mathrm{and}\;k=0,1,\ldots ,N_{g}-1.$
$HXY=-\sum_{i}\sum_{j}p\left(i,j\right)log\left(p\left(i,j\right)\right)$	
$HXY1=-\sum_{i}\sum_{j}p\left(i,j\right)log\left\{p_{x}\left(i\right)p_{y}\left(j\right)\right\}$	
$HXY2=-\sum_{i}\sum_{j}p_{x}\left(i\right)p_{y}\left(j\right)log\left\{p_{x}\left(i\right)p_{y}\left(j\right)\right\}$	
